# Itching and its related factors in subtypes of eczema: a cross-sectional multicenter study in tertiary hospitals of China

**DOI:** 10.1038/s41598-018-28828-6

**Published:** 2018-07-17

**Authors:** Xin Wang, Linfeng Li, Xiaodong Shi, Ping Zhou, Yiwei Shen

**Affiliations:** 1 Department of Dermatology, Beijing Shijitan Hospital, Capital Medical University, 10 Tie Yi Road, Haidian District, Beijing, 100038 China; 2 Department of Dermatology, Beijing Friendship Hospital, Capital Medical University, 95 Yong An Road, Xicheng District, Beijing, 100050 China; 3 Market Research Department, China Telecom Corporation Limited Beijing Research Institute, Beijing, China

## Abstract

Itching is a leading symptom of eczema or dermatitis and has a great impact on patients’ lives. Previous studies on itching have focused mostly on atopic dermatitis (AD). A cross-sectional multicenter study was conducted among outpatients with eczema from 39 tertiary hospitals in mainland China from July 1 to September 30, 2014. This work elaborates on itching in different types of eczema. Itching was very common (97%, 8499/8758) in outpatients with eczema. The severity of the itch increased with age and disease duration (*P* < 0.001). The top three subtypes of dermatitis with severe itching were atopic dermatitis (30.4%), widespread eczema (30.1%), and asteatotic eczema (27.9%). Widespread eczema refers to the involvement of more than three body parts, without clinical features of other specific types of eczema. The proportion of outpatients without itching was highest in hand eczema (6.8%). Positive correlations were observed between the severity of itching and the proportions of different diseases based on trend tests, including atopic dermatitis (*P* < 0.001), widespread eczema (*P* < 0.001), asteatotic eczema (*P* < 0.001), and autosensitization dermatitis (*P* < 0.001). Eczema outpatients with older age, longer disease duration, and, especially, a history of allergic diseases might be more prone to itching.

## Introduction

Pain and itching play critical roles in protecting organisms from sources of danger, such as extreme temperatures, reactive chemicals, and tissue injury. These sensations can result in neuronal modulation of the immune system or behavioral avoidance of future exposure to pathogens. Parasitic (e.g., Onchocerca volvulus) and fungal skin infections (e.g., Tinea pedis, Candida albicans) are characterized by intense itching^1^, which is a common symptom of eczema or dermatitis and has a great impact on patients^2,3^. Various substances that cause itching (pruritogens), such as cytokines and chemical messengers, are released from the affected area^4,5^. The prevalence of itching (once as reported to be approximately 10%) in the general population is not well documented^6^. A cross-sectional study of 2076 adults from a general internal medicine clinic has found that the prevalence of itching was 39.9% and increased with age (from 33.1% at age 19–39 years to 45.9% at age ≥80 years) in the United States^7^. A German population-based cross-sectional study found that the self-reported, 12-month, and lifetime prevalences of chronic pruritus (lasting ≥6 weeks) were 13.5%, 16%, and 22%, respectively^8^. In a Norwegian population-based cross-sectional study on self-reported skin morbidity, pruritus, the dominant symptom among adults, was experienced within the past week by 9% of women and 7.5% of men^9^.

Many studies on itching have been carried out in atopic dermatitis (AD), but very few have focused on other types of eczema. A recent study on AD has reported the frequency (85%), duration (41.5% with itching ≥18 h/d), and severity (6.5 of 10 on a numeric rating scale) of itching, as well as the frequency of AD-related sleep disturbance (55% with disturbance 5 days/week or more)^10^. Itching has significant adverse effects on patients’ quality of life, which can trigger depression^11^, anxiety, sleeplessness, and even suicidal thoughts, indicating more attention should be paid to this symptom. Itching and its related factors in eczema and dermatitis in China are unclear, so we performed a hospital-based multicenter cross-sectional epidemiologic survey in Chinese outpatients with dermatitis and eczema. The purpose of the current study is to examine the association of itching with eczema and dermatitis in a department of dermatology in a Chinese population.

## Results

### Demographic characteristics (N = 8758)

**Figure 1:**
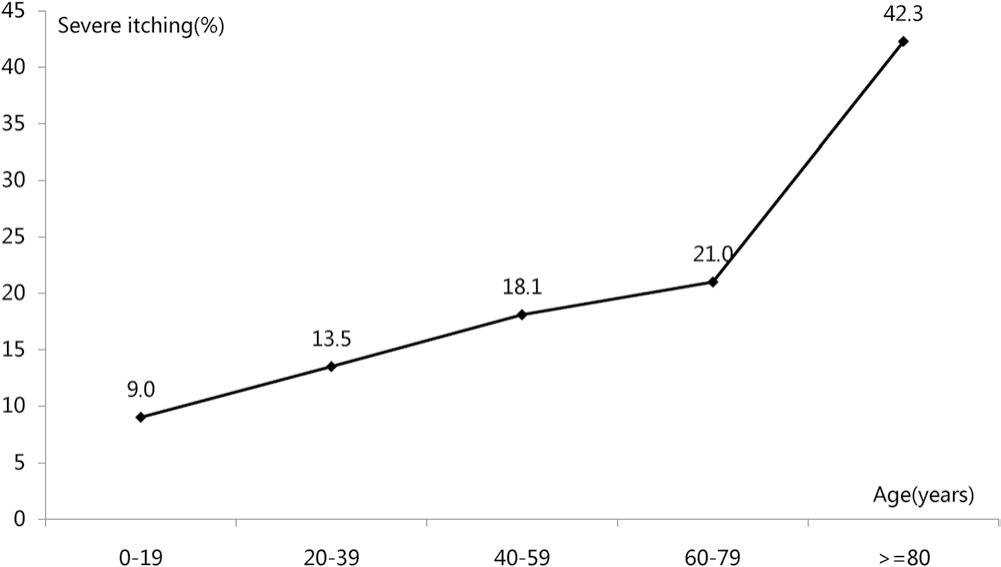
Relationship between age and severe itching. Wilcoxon W Test, *P* < 0.001.

In this survey, 9688 outpatients were approached, and 295 refused to participate in this study (response rate 97%). In total, 9393 outpatients were screened, of whom 636 (6.7%) were excluded because of incomplete information. Finally, 8758 outpatients were recruited, and 97% of them had itching (8499/8758). The basic characteristics of the eczema outpatients with itching are shown in Table 1. The correlation between sex and itching severity was not significant (*P* = 0.069, linear-by-linear association chi-square test). The average age of outpatients with severe itching was 42.06 ± 18.70 years. The 8758 outpatients were divided into five age groups. The proportions of severe itching in these groups were 9% (0–19 years, 145/1604), 13.5% (20–39 years, 489/3629), 18.1% (40–59 years, 433/2397), 21% (60–79 years, 217/1031), and 42.3% (≥80 years, 41/97). A positive correlation between age and severe itching was observed (Wilcoxon W test, *P* < 0.001, Fig. 1). The territory of China is approximately 9.6 million square kilometers; the climate varies greatly with different geographical locations. The association between itching and latitude was investigated, but the results were inconclusive at the current stage.

**Table 1:** Clinical characteristics of eczema outpatients with itching (N = 8758).

Itching Grade^※^					
Variable	No (n = 259)	Mild (n = 2629)	Moderate (n = 4545)	Severe (n = 1325)	*P*
Age (years) (mean ± SD)^a^	32.2 ± 18.46	34.9 ± 17.67	35.3 ± 18.92	42.06 ± 18.70	<0.001
Sex (n, %)^b^					0.069
Male	127 (2.9)	1326 (29.9)	2263 (51.0)	719 (16.2)	
Female	132 (3.1)	1303 (30.1)	2282 (52.8)	606 (14.0)	
Disease duration (years) (mean ± SD)^c^	1.8 ± 2.37	2.3 ± 3.76	3.1 ± 5.53	4.1 ± 5.85	<0.001
Suspected bacterial infection (yes) (n, %)^d^	31 (12.0)	190 (7.2)	441 (9.7)	547 (41.3)	<0.001
History of allergic disease (yes) (n, %)^e^	24 (9.3)	199 (7.6)	717 (12.8)	345 (26.0)	<0.001
History of asthma (yes) (n, %)	3 (1.2)	40 (1.5)	123 (2.7)	61 (4.6)	<0.001
History of allergic rhinitis (yes) (n, %)	7 (2.7)	60 (2.3)	217 (4.8)	117 (8.8)	<0.001
History of allergic conjunctivitis (yes) (n, %)	7 (2.7)	46 (1.7)	187 (4.1)	78 (5.9)	<0.001
History of atopic dermatitis (yes) (n, %)	3 (1.2)	22 (0.8)	65 (1.4)	25 (1.9)	0.007
History of dry skin (yes) (n, %)^f^	49 (18.9)	408 (15.5)	957 (21.1)	476 (35.9)	<0.001
History of flexion dermatitis(yes) (n, %)^g^	33 (12.7)	207 (7.9)	394 (8.7)	244 (18.4)	<0.001
History of infantile eczema(yes) (n, %)^h^	16 (6.2)	145 (5.5)	484 (10.6)	174 (13.1)	<0.001

^a^F = 56.2, one-way ANOVA.

^b^Linear-by-linear association chi-square test.

^c^Jonckheere-Terpstra test.

^d,e,f,g,h^Linear-by-linear association chi-square test.

^※^No: no itching; Mild: neither the participant’s daily activities nor sleep was interrupted; Moderate: daily activities were interrupted, but sleep was not affected; Severe: both daily activities and sleep of participants were affected.

Questions included:

How old are you?

What is your sex?

How long have you suffered from this disease?

Is there a suspected bacterial infection? (diagnosed by a doctor)

Is there a personal and/or family (first-degree relatives) history of atopic diseases (asthma; allergic rhinitis; allergic conjunctivitis; atopic dermatitis)?

Is there a history of a generalized dry skin?

Is there a history of infant eczema?

Is there a history of flexural involvement?

### Clinical characteristics (N = 8758)

The clinical characteristics of eczema outpatients with itching are shown in Table 1. A positive correlation was observed between itching severity and suspected bacterial infection (*P* < 0.001, linear-by-linear association chi-square test). Similarly, a positive history of allergic disease, dry skin, flexion dermatitis and infantile eczema were more common as itching worsened (*P* < 0.001, linear-by-linear association chi-square test).

**Figure 2:**
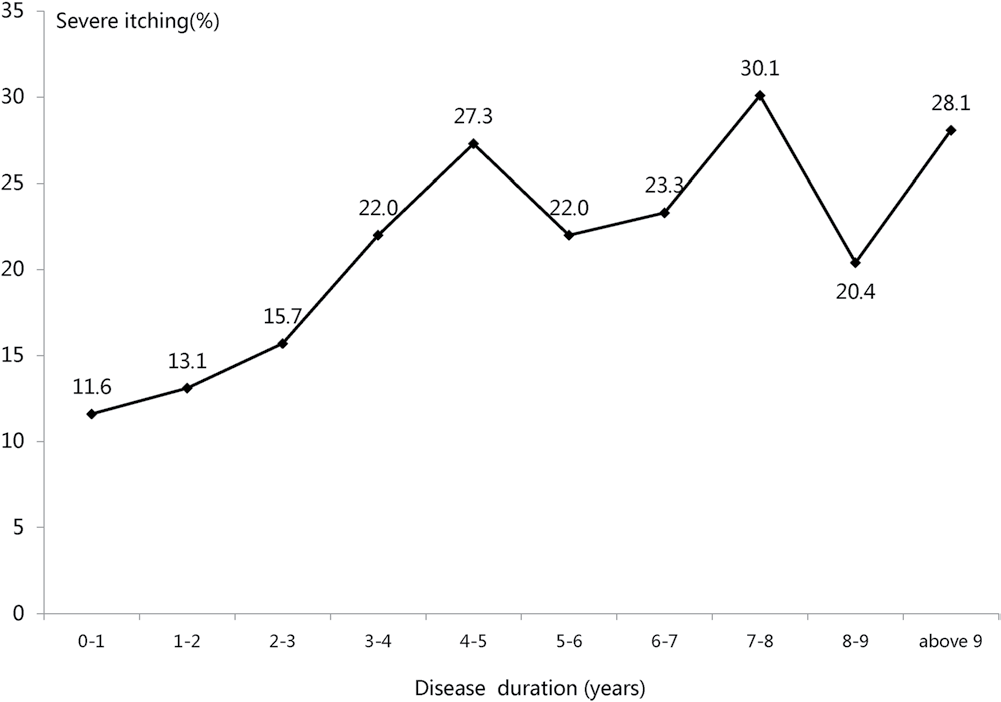
Relationship between disease duration and severe itching. A total of 8758 outpatients were divided into ten groups according to disease duration: the disease durations 9 years or less were divided into single years, and disease durations more than 9 years were combined in a single group. We discovered that the proportion of eczema outpatients with severe itching increased significantly with longer disease duration (Spearman’s rank correlation test, *P* < 0.001).

**Figure 3:**
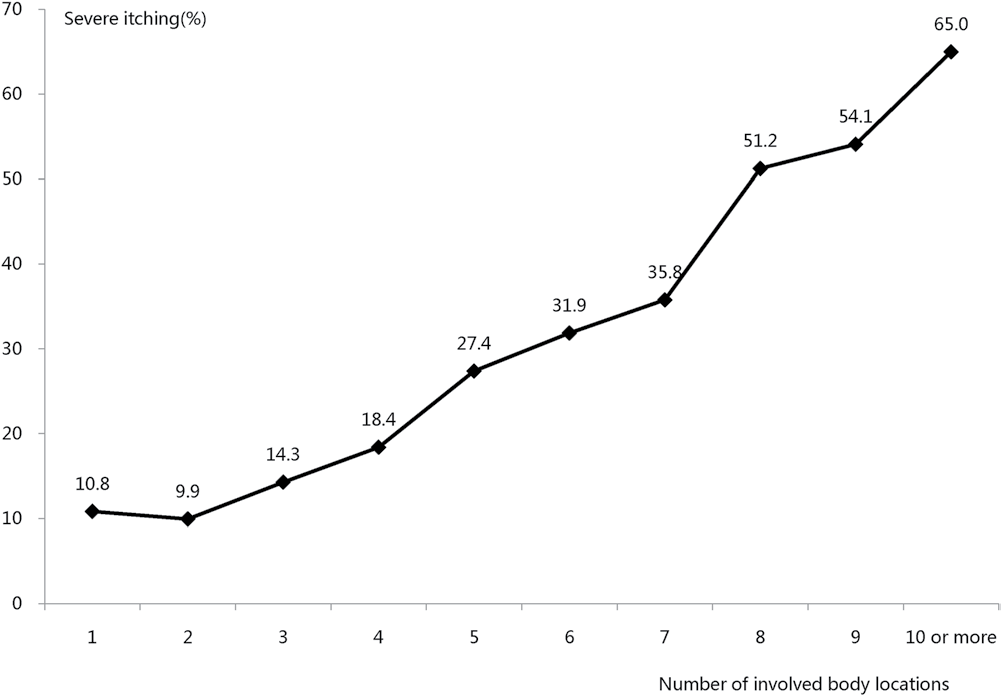
Relationship between the number of involved body locations and severe itching. To analyze the relationship between the number of involved body locations and severe itching, all 8758 patients were divided into 10 groups based on the number of involved body locations, from 1 to ≥10; 1 meant the patient had one body location involved, and 10 or more meant the patient had ten or more body locations involved. Because few patients had more than ten body locations involved, there was no separate group for 11 and 12. We analyzed the proportion of severe itching in each group and found that as the number of involved body locations increased, the proportion of eczema outpatients with severe itching increased significantly (R = 0.988, P < 0.001, Spearman correlation).

**Figure 4:**
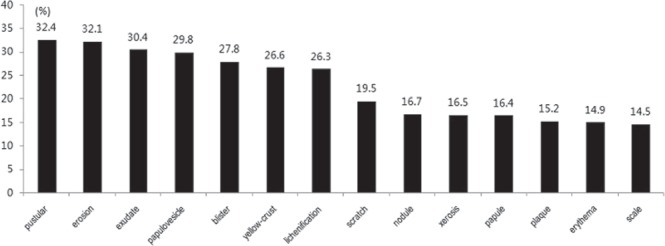
Skin lesion types of eczema outpatients with severe itching (%).

The mean disease duration of the outpatients with severe itching was 4.1 ± 5.85 years. A total of 8758 outpatients were divided into ten groups according to disease duration: the disease durations 9 years or less were divided into single years, and disease durations more than 9 years were combined in a single group. We discovered that the proportion of severe itching in eczema outpatients increased significantly with disease duration (Spearman’s rank correlation test, *P* < 0.001, Fig. 2). As the number of involved body locations increased (range, 1–10), the proportion eczema outpatients with severe itching increased significantly (R = 0.988, *P* < 0.001, Spearman correlation) (Fig. 3). The top three involved sites with severe itching were the vulva (34.4%), chest (33.3%) and axilla (27.5%) (see Supplementary Fig. S1). The most common types of skin lesions with severe itching were pustular (32.4%), erosion (32.1%) and exudate (30.4%) (Fig. 4).

### Pruritus proportion

T he proportion of itching in each type of dermatitis is shown in Table 2. T he top three subtypes of dermatitis with severe itching were atopic dermatitis (30.4%), widespread eczema (30.1%) and asteatotic eczema (27.9%). Widespread eczema refers to the involvement of more than three body parts, without clinical features of other specific types of eczema. T he proportion of outpatients without itching was highest in hand eczema (6.8%).

**Table 2:** T he proportion of itching in each type of dermatitis (N = 8758).

Itching Grade					
Classification^※^	No. of patients (%, n)	Mild (%, n)	Moderate (%, n)	Severe (%, n)	*P* ^*#*^
Unclassified eczema (3109/8758)	2.3 (71)	30.6 (950)	52.5 (1633)	14.6 (455)	0.763
Atopic dermatitis (682/8758)	0 (0)	17.4 (119)	52.2 (356)	30.4 (207)	<0.001
Irritant contact dermatitis (810/8758)	4.8 (39)	34.9 (283)	48.9 (396)	11.4 (92)	<0.001
Widespread eczema (765/8758)	1.8 (14)	18.6 (142)	49.5 (379)	30.1 (230)	<0.001
Hand eczema (590/8758)	6.8 (40)	24.2 (143)	57.5 (339)	11.5 (68)	0.06
Allergic contact dermatitis (513/8758)	2.9 (15)	27.7 (142)	49.5 (254)	19.9 (102)	0.021
Neurodermatitis (483/8758)	2.1 (10)	30.0 (145)	47.3 (228)	20.7 (100)	0.021
Seborrheic dermatitis (447/8758)	3.4 (15)	44.7 (200)	45.0 (201)	6.9 (31)	<0.001
Nummular eczema (400/8758)	1.8 (7)	25.8 (103)	55.8 (223)	16.8 (67)	0.019
Asteatotic eczema (301/8758)	5 (15)	22.9 (69)	44.2 (133)	27.9 (84)	<0.001
Photosensitive dermatitis (244/8758)	2.9 (7)	24.6 (60)	57.0 (139)	15.6 (38)	0.187
Autosensitization dermatitis (221/8758)	1.4 (3)	25.8 (57)	45.7 (101)	27.1 (60)	<0.001
Dyshidrotic eczema (220/8758)	2.3 (5)	29.5 (65)	51.4 (113)	16.8 (37)	0.465
Stasis dermatitis (123/8758)	0.8 (1)	15.4 (19)	69.1 (85)	14.6 (18)	0.289

^※^Dermatitis and eczema were classified based on the International Classification of Diseases (ICD)−10 (eczema ICD-10 codes: L30.902)^33^. T he doctor diagnosed the disease strictly according to the definition of the disease Andrews’ Diseases of the Skin: Clinical Dermatology tenth edition, simplified Chinese edition. T he remaining unspecified eczema was diagnosed as unclassified eczema (UE)^34^. According to the questionnaire and physicians’ evaluation, a comprehensive diagnosis of atopic dermatitis was made based on “UK Working Party criteria”^35^. T he diagnosis was not unique, and no laboratory test was performed for diagnosis. In the cases with overlaps, the diagnosis was made based on medical history and clinical features with high accuracy. In 612 patients, eczema only affected the hands as the most common type. T he five most common types were hand eczema (61.1%), dyshidrotic eczema (9.2%), chronic eczema (8%), allergic contact dermatitis (5.2%), and irritant-contact dermatitis (5.1%). T he proportion of these 5 types represented approximately 88.6% of all hand eczema. T hese results faithfully reflect the actual circumstances in tertiary hospitals of China, which is convenient for appraisal and comparison. ^#^Linear-by-linear association test.

Based on trend tests for itching rank in each type of dermatitis, a positive correlation was observed between the severity of itching and proportion of different diseases, including atopic dermatitis (*P* < 0.001), widespread eczema (*P* < 0.001), allergic contact dermatitis (*P* = 0.021), neurodermatitis (*P* = 0.021), nummular eczema (*P* = 0.019), asteatotic eczema (*P* < 0.001), and autosensitization dermatitis (*P* < 0.001).

### Multivariable logistic regression analysis of itching and related factors (N = 8758)

T he 8758 outpatients were analyzed, and 8499 of them had itching. In the multivariate logistic regression analysis of itching and related factors, independent variables included age, sex, disease duration, suspected bacterial infection, and history of allergic disease, dry skin, infantile eczema, and flexion dermatitis. T he results are listed in Supplementary Table S1.

Based on the multivariate logistic regression analysis, age (OR = 1.011, 95% CI: 1.004–1.018, *P* = 0.003), disease duration (OR = 1.096, 95% CI: 1.039–1.156, *P* = 0.001) and history of allergic disease (OR = 1.667, 95% CI: 1.063–2.616, *P* = 0.026) were positively associated with the risk of itching. A history of flexion dermatitis (OR = 0.496, 95% CI: 0.325–0.758, *P* = 0.001) was negatively associated with itching (but not sex, suspected bacterial infection, or a history of dry skin or infantile eczema).

## Discussion

T he probability of itching occurrence in eczema outpatients is related to many factors including age, disease duration, history of allergic disease and flexion dermatitis. Findings from the age and disease-duration subgroup analyses show that the severity of itching increases with age and disease duration. Age increases the risk of impaired skin barrier function, which can precipitate skin breakdown^12^. Loss of function or structural stability in skin proceeds unavoidably as individuals age, resulting from intrinsic and extrinsic stimuli. T his in turn contributes to a progressive loss of skin integrity. Intrinsic aging proceeds at a genetically determined pace, primarily attributed to the accumulation of damage generated by cellular metabolism as well as biological aging. Extrinsic insults from the environment add to the dermatological signs of aging^13^. Aged skin is frequently characterized by xerosis and pruritus (itching). For patients with severe itching, scratching is almost inevitable. Scratching can aggravate dermatitis. T his vicious feedback loop is called the itch-scratch cycle. Once the itch-scratch cycle is established, conscious effort is no longer sufficient to control scratching. T he act of scratching becomes habitual, so that the disease progresses^14^. Lack of attention to this aspect almost always results in suboptimal treatment. Since the scratching occurs at the subconscious level or while asleep, the patient is powerless to break the cycle by him/herself^15^. Itching is more severe with the extension of the disease course, which is in line with our results.

In the present study, eczema outpatients with suspected bacterial infection are more prone to severe itching. Microbial infection caused by different types of pathogens often exhibit intense pain or itch. T he molecular mechanism responsible for pathogenic infection-induced itching is not well understood. Itching may be directly triggered by pruriceptors or neurons, which are stimulated by pathogens or inflammatory factors released by mast cells^1^. A study of acute lesions of AD patients indicates that S. aureus can easily penetrate or invade the skin, mainly through scratching and feed off skin exudates^16^. T hat study also found that eczema outpatients with pustular, erosion or exudate were more susceptible to severe itching, caused by bacterial infection. T hat means eczema outpatients with erosion or exudate were more susceptible to bacterial infection, which correlated with the severity of itching. Elongation of sensory nerves in the epidermis under the stratum corneum due to drying and inflammation is also considered to be a cause of skin hyperesthesia^13^, which means these patients are more prone to itching. T his suggests the importance of treating bacterial infection in pruritus patients.

A positive association has been identified between itching and history of allergic disease. History of allergic disease included asthma, allergic rhinitis, allergic conjunctivitis and atopic dermatitis (AD). Atopic dermatitis is an inflammatory skin disease characterized by intense pruritus and relapsed eczematous lesions^17^. It increases the risk for food allergy, asthma, allergic rhinitis, immune-mediated diseases, and mental disorders^18^. Specific defects in immune system can cause primary disturbances in immunologic disorders that induce IgE-mediated sensitization, epidermal barrier dysfunction, and subsequent local inflammation^19^. As the most important symptom of AD, pruritus has an abnormal interaction between immune cells and keratinocytes. Dysfunction of these cells activates mediators that enhance the sprouting of nerve fibers and stimulate sensory nerve endings^20^. To promote itch, TSLP derived from keratinocytes communicates directly with cutaneous sensory neurons^21^. TSLP stimulates T h2 cells to produce cytokines, such as IL-13, to strengthen the growth of nerve fibers^22^. T herefore, decreased expression of TSLP and IL-13 is crucial to ameliorate pruritus in AD^17^. We observed a negative association between itching and history of flexion dermatitis. However, the reason is unclear, which requires further exploration.

Our study showed that the top three subtypes of dermatitis with severe itching were atopic dermatitis (30.4%), widespread eczema (30.1%) and asteatotic eczema (27.9%), which was featured by dry skin or large areas of lesions. Based on trend tests for itching rank, positive correlations were observed between the severity of itching and the proportions of these three diseases (*P* < 0.001), as shown in Table 2. Dry skin is characterized by a disrupted barrier, resulting in roughness, desquamation, lack of brightness of the skin surface and development of pruritus^23^. T-cells directly communicate with nerves to regulate neurogenic inflammation of pain and are involved in pruritus as well^24–26^. Recent reports propose that T-cell signaling pathways are involved in dry skin pruritus. Zeta-chain-associated protein kinase 70 (ZAP70), as a T-cell receptor, may induce interleukin 2 (IL-2) secretion and promote nerve growth factor (NGF) secretion in skin. Another study showed that increased ZAP70 is involved in dry skin in elderly pruritus, while increased secretion of IL-2 and NGF may induce dry-skin itching^27^. Dry skin is one of the major causes of itching. T herefore, the European Guideline on chronic pruritus recommends emollients as the first-line therapy to restore the skin barrier and to obtain relief of pruritus^28^.

Several limitations should be considered when interpreting our results. Since itching was assessed mainly based on the subjective experience of outpatients, quantitative analysis of itching in patients with itching dermatitis is indispensable for disease status and response to therapy^29^. However, we did not use a standardized checklist, such as scoring atopic dermatitis (SCORAD), Nottingham eczema severity score (NESS), or dermatology life quality index (DLQI). Our questionnaire was based on the “UK working Party criteria” (UKWP), which has a high specificity (≥90%) but low sensitivity (40–100%). A recent study in Taiwan shows that UKWP criteria could serve as a better indicator for identifying adult AD than its counterpart, the International Study of Asthma and Allergies in Childhood (ISAAC)^30^. However, that study reported a 4.3% difference in AD prevalence from the use of UKWP criteria (3.7%) vs. dermatologists’ diagnosis (8.0%). Intriguingly, similar findings were observed in a Japanese investigation, in which the prevalence of adult AD was estimated to be approximately 2.9% using the UKWP criteria, compared to 6.9% when diagnosed by the dermatologists^31,32^. T hese results suggested that UKWP criteria underestimate the true prevalence of adult AD. In 2016, Professor Zhang proposed three features as the criteria for adult/adolescent AD; of all 2662 patients, 60.3% satisfied the Chinese criteria, 48.2% satisfied the Hanifin and Rajka criteria, and 32.7% satisfied the Williams criteria^33^. T he Chinese criteria have higher sensitivity for adult/adolescent AD patients. Laboratory tests are necessary, but not all patients agree to do it. T he UKWP criteria are suitable for epidemiological study, but some AD patients might be missed according to the Chinese criteria.

Other major limitations of this study include the following. Because participants were recruited from multiple tertiary referral hospitals located in provincial capitals or central cities, most patients visiting these hospitals were in a better financial and medical-insurance status than the average. As a hospital-based study, a selective bias was inevitable due to a nonhomogeneous population and differential spatial distribution. In addition, no adequate treatment or follow-up were documented in our study. All these factors may have led to an unavoidable bias.

In summary, this study provides an informative profile of itching in Chinese outpatients. Eczema outpatients with older age, longer disease duration, and, especially, a history of allergic diseases might be more prone to itching. T hese results provide important evidence for the prevention and therapy of itching. High-quality cohorts or case-control studies are needed to verify the results of this survey.

## Methods

### Study design and subjects

T his study was approved by Institutional Review Board (IRB) committee of Beijing Friendship Hospital, Capital Medical University. All experiments were performed in accordance with relevant guidelines and regulations. All participants provided oral informed consent. T his prospective and cross-sectional study was conducted from July 1 to September 30, 2014. T he information of outpatients diagnosed with eczema and dermatitis were collected from 39 tertiary hospitals in 15 provinces and municipalities in mainland China, including Guangdong, Chongqing, Hunan, Jiangxi, Henan, Zhejiang, Shanghai, Hubei, Jiangsu, Anhui, Shanxi, Beijing, Tianjin, Shandong, and Liaoning provinces, which covered most areas of China^34^ (see Supplementary Fig. S2). T he inclusion criteria were outpatients diagnosed with eczema and dermatitis visiting the 39 tertiary hospitals from July 1 to September 30, 2014. T he exclusion criteria included outpatients with serious mental illness or organic disease who could not cooperate with the investigation and outpatients who refused to provide oral informed consent.

### Specific research content

All enrolled outpatients completed a specific survey on general demographic characteristics, disease duration, severity of itching, distribution of lesions, type of skin lesions and medical history. Itching was assessed and divided into four levels: (i) no itching; (ii) mild itching (neither the participant’s daily activities nor sleep was interrupted); (iii) moderate itching (daily activities were interrupted, but sleep was not affected); and (iv) severe itching (both the daily activities and sleep of participants were affected) (see Supplementary Fig. S3). History of allergic disease, dry skin, infantile eczema, and flexion dermatitis were also recorded. History of allergic disease included asthma, allergic rhinitis, allergic conjunctivitis and AD. Secondary bacterial infection was clinically suspected if superficial pustules, prudent exudation, or yellow crust was detected. Our previous study has shown such lesions correlated with laboratory results of bacterial culture. In eczema with clinical diagnosed bacterial infection, *S. aureus* was isolated in 92.9% of patients^35^.

### Diagnostic criteria

All dermatologists involved in this study had abundant experience in clinical diagnosis and treatment of eczema and were trained in a standardized manner before the project. First, each subject was inspected by a dermatologist independently. T hen, a questionnaire survey was conducted by dermatologists after a 10–15-minute dermatological physical examination and after the parents or guardians had filled out informed consent forms for pediatric outpatients.

Dermatitis and eczema were classified based on the International Classification of Diseases (ICD)−10 (eczema ICD-10 codes: L30.902)^36^. T he doctor diagnosed the disease strictly according to the definition of the disease from Andrews’ Diseases of the Skin: Clinical Dermatology tenth edition, simplified Chinese edition. Specific types of dermatitis, including atopic dermatitis (AD), irritant contact dermatitis (ICD), widespread eczema, hand eczema (HE), allergic contact dermatitis (ACD), neurodermatitis, seborrheic dermatitis, nummular eczema, asteatotic eczema, photocontact dermatitis, autosensitization eczema, dyshidrotic eczema, and stasis dermatitis, were clinically diagnosed accordingly. T he remaining, unspecified eczema was diagnosed as unclassified eczema (UE)^37^. According to the questionnaire and physicians’ evaluation, a comprehensive diagnosis of AD was made based on “UK Working Party criteria”^38^. Multiple diagnoses were possible, and no laboratory test was performed for diagnosis.

### Statistical Analysis

All data were input into Statistical Package of Social Sciences software version 17.0 (IBM, NY) for statistical analysis. For continuous variables, mean ± standard deviation (SD) was used according to distribution. T he normality of data was checked by the Kolmogorov-Smirnov test. Age conformed to a normal distribution, but disease duration was characterized by a skewed distribution. Statistical methods included the t-test, chi-square test, and correlation analysis. Differences in age and disease course between itching grade groups were evaluated by one-way ANOVA. Differences in sex, medical history, type of dermatitis and suspected bacterial infection between itching grade groups were analyzed by the chi-square tests. Missing data were excluded from the final analyses. All the analyses were two-tailed tests with the significance level of 0.05. Age, sex, disease duration, suspected bacterial infection, and history of allergic disease, dry skin, infantile eczema, and flexion dermatitis were included in logistic regression models for odds ratios (ORs) and 95% CI estimation.

## Electronic supplementary material

Supplementary Information
