# Supplementary material for: Itching and its related factors in subtypes of eczema: a cross-sectional multicenter study in tertiary hospitals of China
Source: Sci Rep. 2018 Jul 17;8:10754. doi: 10.1038/s41598-018-28828-6 (PMC6050257; doi:10.1038/s41598-018-28828-6)
Supplement: Supplementary file 1 — Supplementary Information [file 41598_2018_28828_MOESM1_ESM.pdf]

**Itching and its related factors in subtypes of eczema: a cross-sectional multicenter study in tertiary hospitals of China**

Xin Wang[1], Linfeng Li * [2], Xiaodong Shi[3], Ping Zhou[1], Yiwei Shen[1]

[1]Department of Dermatology, Beijing Shijitan Hospital, Capital Medical University. Department of Dermatology, Beijing Shijitan Hospital, Capital Medical University, 10 Tie Yi Road, Haidian District, Beijing, 100038, China.

[2]Department of Dermatology, Beijing Friendship Hospital, Capital Medical University.
Department of Dermatology, Beijing Friendship Hospital, Capital Medical University, 95 Yong An Road, Xicheng District, Beijing, 100050, China.

[3]Market Research Department, China Telecom Corporation Limited Beijing Research Institute.

**Corresponding author**

Lin-Feng Li, Department of Dermatology, Beijing Friendship Hospital, Capital Medical University, 95 Yong An Road, Xicheng District, Beijing 100050, P.R. China

Email: zoonli@sina.com;Tel:86-013693620186.

Supplementary Fig. S1. Lesions distribution of eczema outpatients with severe itching (%)

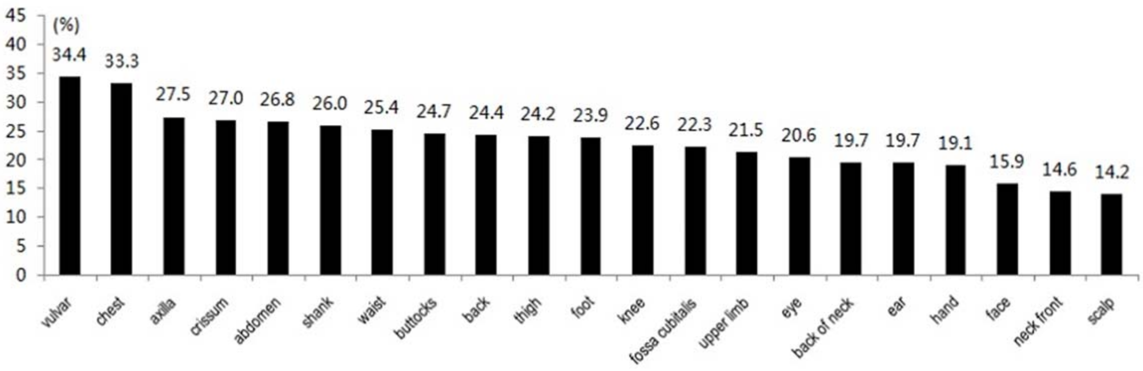

Supplementary Fig. S2. Geographic distribution of the investigated hospitals.

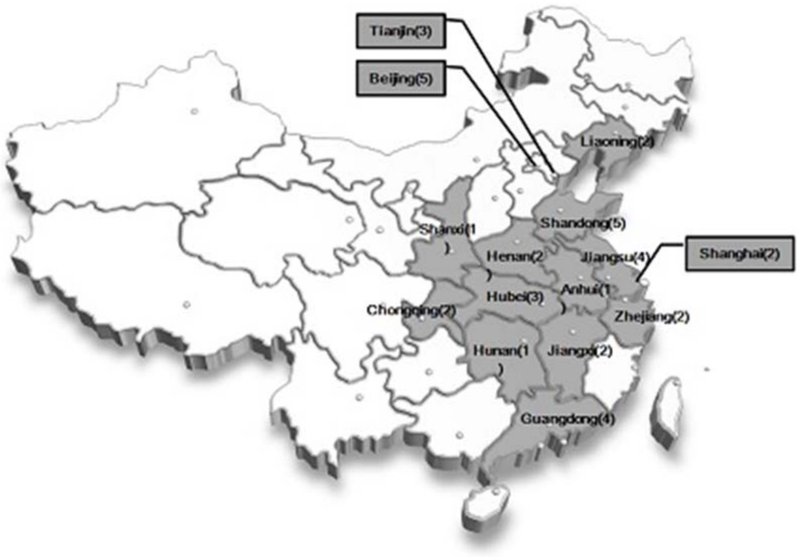


The shaded part indicates the provinces investigated in this study, and the parenthetical figure is the number of hospitals participating in the survey. The figure has been developed using computerized Tube Map4.1 edition software by author (http://www.maptube.org/).

Supplementary Fig. S3. A patient with severe itching

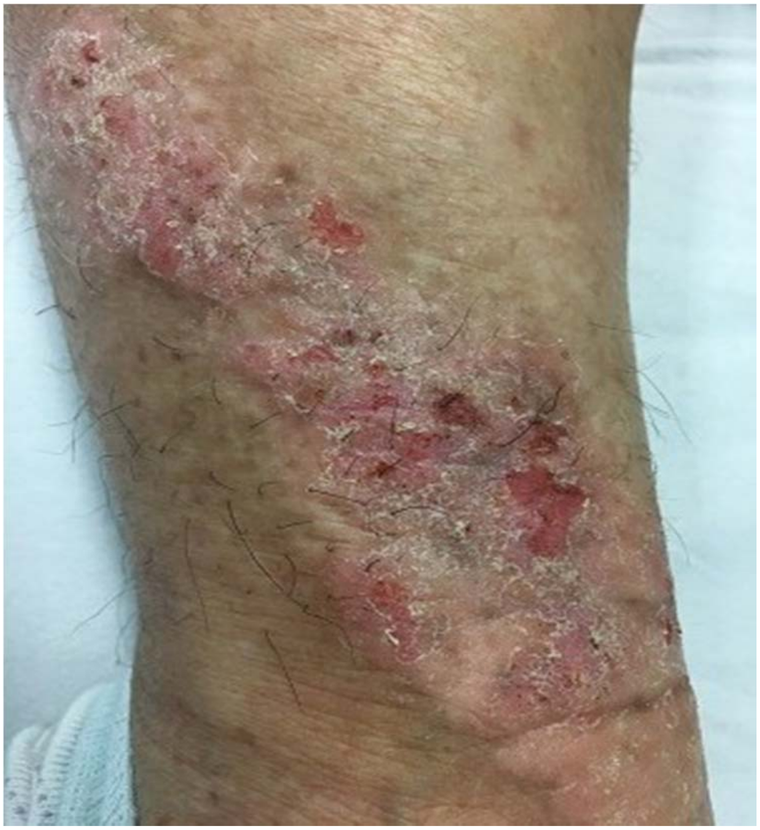

There is a photograph of lower leg with severe itching.

Supplementary Table S1. Logistic regression of itching and related factors (N=8758)

| | *N(%)* | *OR* | *95%CI* | *P* |
|---|---|---|---|---|
| Gender | | | | |
| Male | 4435(50.6) | 1 | | |
| Female | 4323(49.4) | 0.947 | 0.739-1.214 | 0.668 |
| Age | | 1.011 | 1.004-1.018 | 0.003 |
| Disease duration | | 1.096 | 1.039-1.156 | 0.001 |
| Suspected bacterial infection | 1209 (13.8) | 1.087 | 0.736-1.604 | 0.675 |
| History of allergic disease | 1285(14.7) | 1.667 | 1.063-2.616 | 0.026 |
| History of dry skin | 1890(21.6) | 1.024 | 0.718-1.462 | 0.894 |
| History of infantile eczema | 819(9.4) | 1.680 | 0.974-2.899 | 0.062 |
| History of flexion dermatitis | 878(10.0) | 0.496 | 0.325-0.758 | 0.001 |

Questions included: (filled in by doctor)

Age__                Gender

How long have you suffered from this disease?___ year ___ month

Which of the following in accordance with your situation?

(i) no itching;

(ii) mild itching (neither the participant's daily activities nor sleep was interrupted);

(iii) moderate itching (daily activities were interrupted but sleep was not affected);

(iv) severe itching (both daily activities and sleep of participants were affected)

Which body locations were involved?

__ fossa cubitalia, __knee, __vulvar, __chest, __axilla, __crissum,

__abdomen, __shank, __ thigh, __foot, __upper limb, __eye, __back of neck, __ear, hand, __face, __neck front, __scalp, __waist,____buttocks, __back.

Which types of skin lesions were involved?

__erosion, ___exudate, ___papulovesicle, ___blister,___yellow-crust,_lichenification, scratch, _nodule, ___xerosis,___papule, __,pustular.

Is there personal and/or family (the first-degree relative) history of atopic diseases? (asthma; allergic rhinitis; allergic conjunctivitis; atopic dermatitis)__ yes, __no

Is there a history of a generalized dry skin?__ yes, __no

Is there a history of infant eczema?  __ yes, __no

Is there a history of flexural involvement?__ yes, __no

Is there a suspected bacterial infection? (diagnosed by doctor) ___ yes, ___ no

What is the diagnosis? (diagnosed by doctor)

___atopic dermatitis, ___irritant contact dermatitis, ___widespread eczema,

___hand eczema, ___allergic contact dermatitis, ___neurodermatitis, ____seborrheic dermatitis, ___nummular eczema, ___asteatotic eczema, ____photo-contact dermatitis,

autosensitization eczema, ___dyshidrotic eczema, ___stasis dermatitis, ___unclassified eczema.
